# Characterization of *Rice Black-Streaked Dwarf Virus*- and *Rice Stripe Virus*-Derived siRNAs in Singly and Doubly Infected Insect Vector *Laodelphax striatellus*

**DOI:** 10.1371/journal.pone.0066007

**Published:** 2013-06-11

**Authors:** Junmin Li, Ida Bagus Andika, Jiangfeng Shen, Yuanda Lv, Yongqiang Ji, Liying Sun, Jianping Chen

**Affiliations:** 1 State Key Laboratory Breeding Base for Zhejiang Sustainable Pest and Disease Control, Ministry of Agriculture Key Laboratory of Biotechnology in Plant Protection, Institute of Virology and Biotechnology, Zhejiang Academy of Agricultural Sciences, Hangzhou, Zhejiang Province, China; 2 College of Chemistry and Life Science, Zhejiang Normal University, Jinhua, Zhejiang Province, China; 3 Provincial Key Laboratory of Agrobiology, Jiangsu Academy of Agricultural Sciences, Nanjing, Jiangsu Province, China; 4 College of Biotechnology and Life Science, Yangzhou University, Yangzhou, Jiangsu Province, China; University of Kansas Medical Center, United States of America

## Abstract

Replication of RNA viruses in insect cells triggers an antiviral defense that is mediated by RNA interference (RNAi) which generates viral-derived small interfering RNAs (siRNAs). However, it is not known whether an antiviral RNAi response is also induced in insects by reoviruses, whose double-stranded RNA genome replication is thought to occur within core particles. Deep sequencing of small RNAs showed that when the small brown planthopper (*Laodelphax striatellus*) was infected by *Rice black-streaked dwarf virus* (RBSDV) (*Reoviridae*; *Fijivirus*), more viral-derived siRNAs accumulated than when the vector insect was infected by *Rice stripe virus* (RSV), a negative single-stranded RNA virus. RBSDV siRNAs were predominantly 21 and 22 nucleotides long and there were almost equal numbers of positive and negative sense. RBSDV siRNAs were frequently generated from hotspots in the 5′- and 3′-terminal regions of viral genome segments but these hotspots were not associated with any predicted RNA secondary structures. Under laboratory condition, *L. striatellus* can be infected simultaneously with RBSDV and RSV. Double infection enhanced the accumulation of particular genome segments but not viral coat protein of RBSDV and correlated with an increase in the abundance of siRNAs derived from RBSDV. The results of this study suggest that reovirus replication in its insect vector potentially induces an RNAi-mediated antiviral response**.**

## Introduction

The small brown planthopper (*Laodelphax striatellus*; family Delphacidae, order Hemiptera) is one of the most economically important insects and is found world-wide, mainly in temperate regions [1]. The agricultural importance of *L. striatellus* is mainly because it is an efficient vector of two economically important rice viruses: *Rice black-streaked dwarf virus* (RBSDV) and *Rice stripe virus* (RSV) in a persistent propagative manner [2], [3]. RBSDV and RSV move from the insect gut into the hemolymph or other organs and finally enter the salivary glands [4]. Both viruses can replicate in the different organs of the planthopper and are transmitted to plants during feeding [2]–[4]. RBSDV (genus *Fijivirus*, family *Reoviridae*) is the causal agent of rice black-streaked dwarf and maize rough dwarf diseases, which cause severe yield losses in Asia. The RBSDV genome contains 10 segments of dsRNA (S1−S10) which are numbered in decreasing order of size [5]. RSV is the type member of genus *Tenuivirus* and its genome consists of 4 single stranded RNA segments. The complementary strand of RSV RNA1 contains a single open reading frame (ORF) while each of the other segments has two non-overlapping ORFs with ambisense coding strategies separated by a non-coding intergenic region [6]. Co-infection of rice by RSV and RBSDV has been observed in the field [7], but it has not been shown whether the two viruses can be present simultaneously within the same insect vector in nature.

RNA silencing, or RNA interference (RNAi), is an important and conserved pathway to combat virus infections in plants, fungi and insects [8]–[10]. During initiation of the RNAi pathway, double-stranded RNAs (dsRNAs) are recognized by the RNase-III enzyme called Dicer and processed into duplexes of small interfering RNAs (siRNAs) ∼21–24 nucleotides (nt) long [11]. In Drosophila, the three key components involved in this antiviral RNAi pathway are Dicer 2 (DCR2), dsRNA-binding protein 2 (R2D2) and Argonaute 2 (AGO2). Viral dsRNAs or highly structured single-stranded RNAs (ssRNA) are cleaved by DCR2, to produce 21-nt viral-derived siRNA (v-siRNA) duplexes. These v-siRNA duplexes are then recruited by the DCR2-R2D2 complex into RNA-induced silencing complexes (RISCs) containing AGO2, which results in AGO2-mediated specific cleavage of target viral RNAs [12].

The importance of the RNAi pathway for antiviral defence in insects has been clearly demonstrated by genetic studies in Drosophila, where reduced survival rates were observed when DCR2 or AGO2 null mutants were infected with several insect viruses [13]–[15]. Studies using deep sequencing technology have identified the accumulation of siRNAs derived from several RNA viruses in Drosophila [16], [17] and mosquitoes [18]–[21] and from RSV in *L. striatellus* [22], supporting the notion that RNAi is a general antiviral defence in insects. The nature of the viral RNA substrate of Dicer used to generate v-siRNAs has been the subject of much investigation. During the course of RNA virus replication, viral dsRNAs, termed dsRNA replicative intermediates (dsRNA RI), are thought to form during the synthesis of both positive (+)- and negative (−)-strand RNAs by the viral RNA-dependent RNA polymerase (RdRP) [23]. Studies on the characteristics of siRNAs derived from ssRNA viruses in insects suggest that v-siRNAs are predominantly generated from viral dsRNA RI [16]–[18], [20], [24]. On the other hand, in the replication of a number of dsRNA viruses, as exemplified by members of the family *Reoviridae*, synthesis of viral mRNA from the (−)-strand genomic RNA occurs inside subviral particles called cores by particle-associated RdRP. Furthermore, after sorting and packaging of viral mRNAs, synthesis of the viral dsRNA genome from viral mRNA templates is thought to occur concomitantly with the assembly of core particles, therefore the dsRNA genomes of the reoviruses are never free in the cytoplasm but always packaged within viral core particles [25], [26]. This intra-particle mechanism of genome replication may protect viral dsRNAs from recognition by the host. Thus, characterization of siRNAs-derived from reoviruses may provide a new insight into the mechanism by which an insect host senses viral dsRNA and generates v-siRNAs.

In this study, the characteristics of RBSDV siRNAs in *L. striatellus* were comprehensively analyzed using data captured by deep sequencing of small RNAs. We also established co-infection of RBSDV with RSV in *L. striatellus* and examined the effect of simultaneous infection on the v-siRNA profiles of both viruses. Our results show that there are abundant RBSDV siRNAs in infected adult planthoppers. Importantly, RBSDV siRNAs were frequently generated from hotspots in the 5′- or 3′-terminal regions of both strands of the viral genome segments. In double infections with RSV, there were increases in accumulation of particular RNA segments and in the abundance of RBSDV siRNAs.

## Results

### Double Infections of RBSDV and RSV in *L. striatellus*

**Figure 1:**
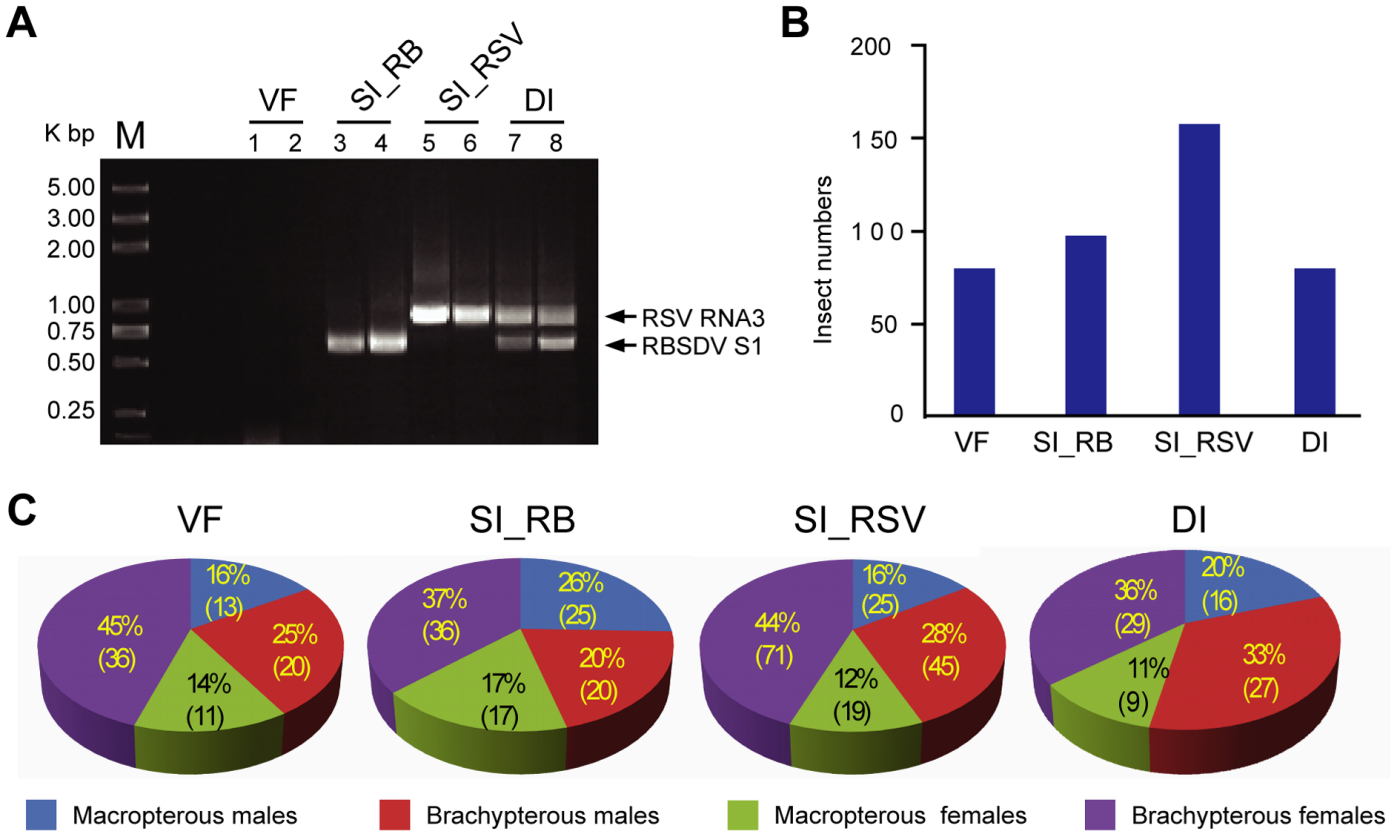
Double infection of RBSDV and RSV in *L. striatellus*. (**A**) RT-PCR analysis of virus accumulation in individual adult insects. An agarose gel electrophoresis showing virus free (VF; lane 1 and 2), RBSDV-infected (SI_RB; lane 3 and 4), RSV-infected (SI_RSV; lane 5 and 6) and doubly-infected (DI; lane 7 and 8) samples. Lengths of predicted PCR products: RBSDV S1, 691 bp; RSV RNA3, 936 bp. (**B**) Numbers of *L. striatellus* carrying single or double viruses or that were virus free from a total of 419 insects analyzed. (**C**) The proportions of *L. striatellus* of different genders or wing form [macropterous (long-winged) or brachypterous (short-winged)] in the insect populations carrying single or double viruses or that were virus free.

Mixed infection of RSV and RBSDV in the insect vector *L. striatellus* has not previously been reported. We therefore took nymphs derived from the RSV-infected *L. striatellus* population and allowed them to feed on RBSDV-infected rice plants. RT-PCR analysis of several individuals of the initial insect population indicated that about 50% (17/30) of them were carrying RSV. After the acquisition period (2 days) and subsequent growth on healthy rice plants (20 days), the presence of viruses in individual adult insects was determined by RT-PCR using RSV- and RBSDV-specific primers. First, we attempted to extract total RNAs from the salivary gland of individual insects, however, very low quantities of RNA were obtained from this organ. Therefore, total RNAs were extracted from whole body of the insect. RT-PCR detection showed that this adult *L. striatellus* population contained a mixture of individual insects that were carrying one or both viruses or that were virus free (Fig. 1A and data not shown). Of 419 insects analyzed, 160 were infected with RSV (SI_RSV) (SI; singly infected), 98 were infected with RBSDV (SI_RB), 81 were doubly-infected (DI) and 80 were virus free (VF) (Fig. 1B). This result demonstrates that *L. striatellus* can carry RSV and RBSDV simultaneously. Additionally, wing form [macropterous (long-winged) or brachypterous (short-winged)] and gender did not significantly influence the virus acquisition (*P* = 0.35 in chi squared test) (Fig. 1C).

### RBSDV siRNAs were Generated in Infected *L. striatellus* and were More Abundant than RSV siRNAs

**Figure 2:**
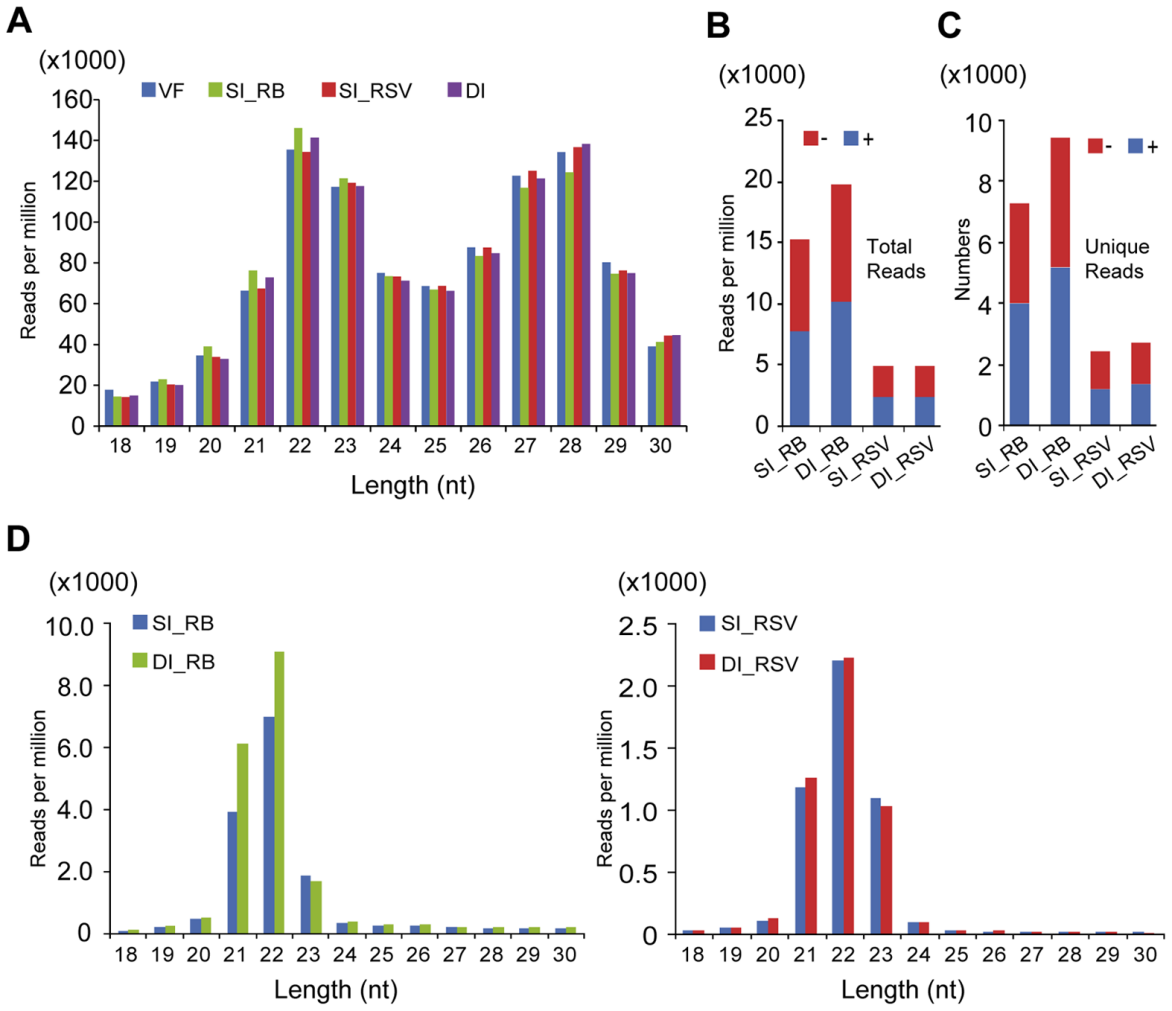
Profiles of *L. striatellus* small RNAs captured by deep sequencing. (**A**) Size distribution of small RNAs derived from virus free (VF), RBSDV-infected (SI_RB), RSV-infected (SI_RSV) and doubly-infected (DI) libraries. (**B**) Abundance of RBSDV and RSV siRNAs derived from singly and doubly-infected libraries. (**C**) Numbers of unique RBSDV and RSV siRNA derived from singly and doubly-infected libraries. (**D**) Size distributions of RBSDV and RSV siRNAs. “−” and “+” indicate siRNAs derived respectively from the complementary (negative) or positive genomic strands. All reads in this analysis are redundant and normalized except (C) which is unique.

Sequencing of the small RNA cDNA libraries from RBSDV-infected, RSV-infected, doubly-infected and virus-free insects produced around 3.0 to 4.5 million raw reads for each library (Table 1). The size distribution pattern of small RNAs (normalized reads) of the four libraries similarly showed two peaks around 22- and 28-nt (Fig. 2A), resembling a previous report on the brown planthopper *Nilaparvata lugens* where the small RNAs had peaks around 22- and 26-nt [27]. Thus, the overall size distribution pattern of small RNAs in *L. striatellus* is not affected by virus infection.

**Table 1:** Summary of small RNA deep sequencing results.

Library^a^	Number of total small RNA reads	Number of viral siRNA reads^b^					
		No mismatch^c^		One mismatch		Two mismatches	
		RSV (%) d	RBSDV (%)	RSV (%)	RBSDV (%)	RSV (%)	RBSDV (%)
**VF**	4,543,883	325 (0.01%)	195 (0.00%)	668 (0.01%)	380 (0.01%)	926 (0.02%)	1,509 (0.03%)
**SI_RSV**	4,017,187	10,083 (0.25%)	193 (0.00%)	19,818 (0.49%)	313 (0.01%)	22,986 (0.57%)	1,569 (0.04%)
**SI_RB**	3,250,004	14 (0.00%)	32,434 (1.00%)	26 (0.00%)	49,304 (1.52%)	119 (0.00%)	52,800 (1.62%)
**DI**	3,028,667	7,865 (0.26%)	39,716 (1.31%)	15,080 (0.50%)	59,596 (1.97%)	17,549 (0.58%)	63,310 (2.09%)

a **VF**, virus free; **SI_RSV**, singly-infected with RSV; **SI_RB**, singly-infected with RBSDV; **DI**, doubly-infected with RSV and RBSDV.

b Redundant and non-normalized siRNAs.

c Number of nucleotide mismatch to viral genomes.

d Percentage of total small RNA reads in the corresponding library.

Computational analyses were performed to search for the presence of RBSDV- and RSV-derived siRNAs in each library. Because there are nucleotide sequence variations among RBSDV and RSV isolates, the search was performed allowing for no, one or two mismatches with the virus reference genome sequences. As shown in Table 1, a large number of small RNAs were found to be derived from RBSDV in the SI_RB and DI libraries and from RSV in the SI_RSV and DI libraries. A very small number of small RNAs in the SI_RSV and VF libraries matched the RBSDV genome or from the SI_RB and VF libraries matched the RSV genome (Table 1). Cross contamination of the samples is unlikely because the presence or absence of the viruses in each individual insect was confirmed by RT-PCR. Thus, these matched small RNAs in the VF library appear to be derived from insect genomic regions with high sequence similarity to each virus genome. It has previously been reported that a small percentage of small RNAs can be mapped to both the viral and the host genomes, making the determination of their origin difficult [19], [28]. The search allowing for one mismatch yielded almost twice as many matches to the viral genomes compared with the no mismatch search, but there was little increase when allowing for two mismatches (Table 1). We therefore used the v-siRNA data derived from the one mismatch search for further analyses. Around 75% of the small RNAs in the VF library that matched the genomes of RBSDV and RSV were also present in the SI_RSV, SI_RBSDV and DI libraries (data not shown), and those small RNAs were excluded from further analyses.

When the read numbers of RBSDV and RSV siRNAs were normalized with the total reads of the corresponding libraries, it is evident that siRNAs derived from RBSDV were much more abundant (more than twice) than those from RSV in both singly and doubly-infected libraries (Fig. 2B). When v-siRNAs were counted based on the number of unique siRNAs (not identical in sequence to any other siRNAs), RBSDV siRNAs were also much more abundant than RSV siRNAs (Fig. 2C). RBSDV and RSV siRNAs were almost equally derived from (+)- and (−)-strand viral genomic RNAs in both singly and doubly-infected libraries: the proportions of sense siRNAs for RBSDV were 50.75% and 51.28% in the SI and DI libraries respectively, and correspondingly 48.67% and 48.00% for RSV (Fig. 2B). Most virus-derived siRNAs were 21-, 22- or 23-nt long, with 22-nt being most abundant in both singly and doubly-infected libraries (Fig. 2D). This is consistent with the peak sizes reported recently for RSV siRNAs in *L. striatellus* [22].

### Co-infection with RSV Elevated RBSDV siRNA Abundance and RNA Accumulation Levels

**Figure 3:**
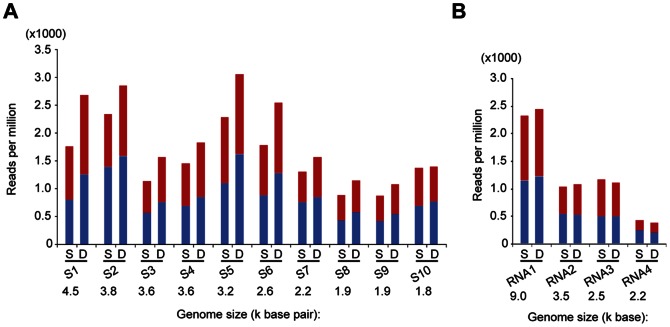
Abundance of viral siRNAs mapped to the different RNA segments of RBSDV (A) and RSV (B). S and D indicate respectively singly and doubly-infected libraries. “−” and “+” indicate siRNAs derived respectively from the complementary (negative) or positive genomic strands. All reads in this analysis are redundant and normalized.

RBSDV siRNAs were markedly more abundant (23% higher) in the DI than in the SI_RB library (Fig. 2B) and this is due to difference in the numbers of 21- and 22-nt RBSDV siRNAs between those libraries (Fig. 2D). Similarly, the number of unique RBSDV siRNAs was markedly higher (23%) in the DI than in the SI_RB library (Fig. 2C), suggesting that the increased abundance of RBSDV siRNAs in doubly infected insects might result from the use of additional Dicer cleavage sites. As shown in Fig. 3A, the abundance of RBSDV siRNAs varied among the ten RBSDV genomic segments and there was no correlation between the segment size and the abundance of siRNAs. For example, RBSDV S5 had the most abundant siRNAs reads, whereas S9 had the least. Most RBSDV genome segments yielded higher numbers of v-siRNA reads in the DI library than in the SI_RB library. The increase was most dramatic with S1, S5, and S6 whereas there were similar read numbers from S10 in both libraries (Fig. 3A).

In contrast, RSV siRNAs read numbers (normalized or unique) were almost identical in the SI_RSV and DI libraries (Fig. 2B and 2C). The abundance of RSV-siRNA reads was correlated with the size of the respective genomic segment, RNA1 having the highest number of siRNA reads and RNA4 the least (Fig. 3B). Furthermore, there were no obvious differences in v-siRNA read numbers among RNA segments between the SI_RSV and DI libraries (Fig. 3B). Thus co-infection of *L. striatellus* with RBSDV and RSV results in increased accumulation of RBSDV siRNAs but has no effect on the accumulation of RSV siRNAs.

**Figure 4:**
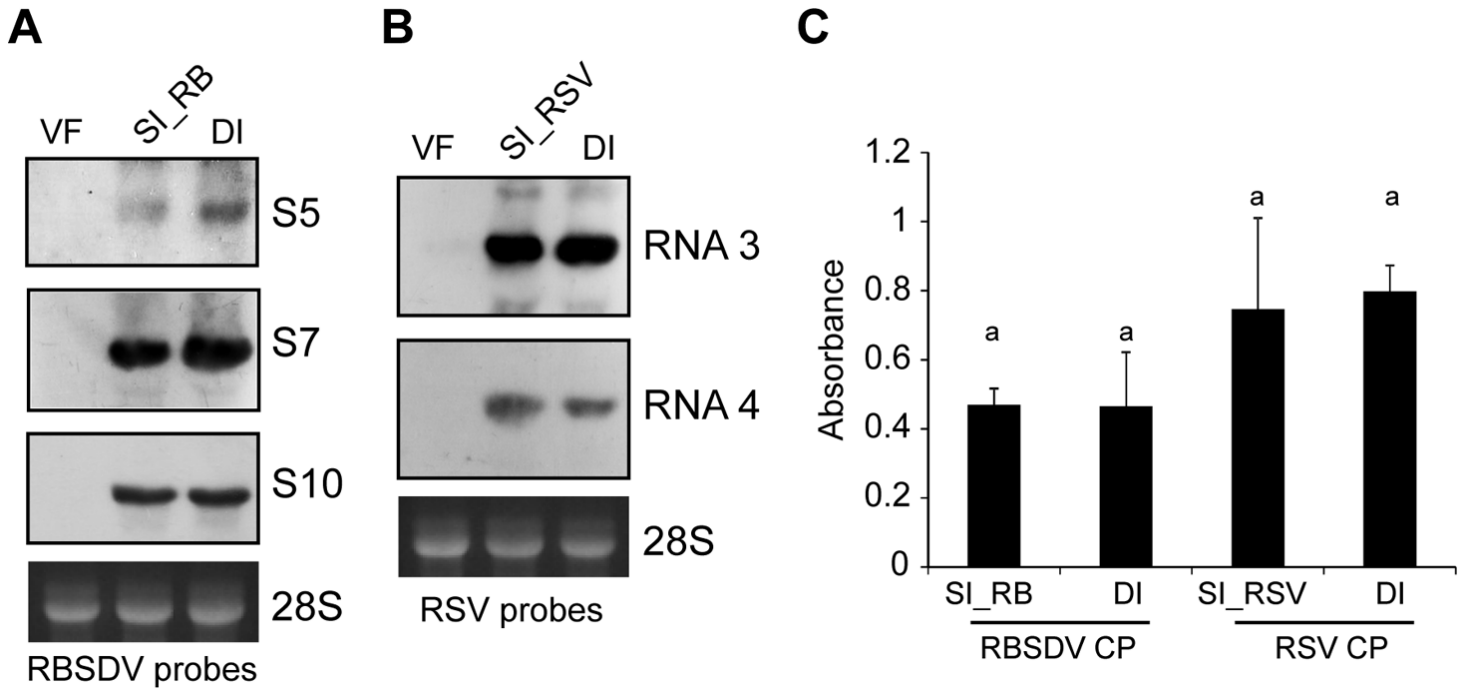
Viral RNA and coat protein accumulation of RBSDV and RSV in *L. striatellus*. (**A** and **B**) Northern blot analysis to compare RNA accumulation levels of RBSDV (**A**) and RSV (**B**) between singly (SI_RB or SI_RSV) and doubly-infected (DI) insects. Ethidium bromide-stained 28S rRNA is shown as loading control (bottom panel). (**C**) Enzyme-linked immunosorbent assay (ELISA) detection of RBDSV and RSV coat protein (CP) accumulation in singly and doubly-infected insects. Similar letters on top of the bars indicate non-significant differences (Student’s t-test) of coat protein accumulation levels between SI_RB and DI or SI_RSV and DI samples.

Next, we investigated whether the increase of RBSDV siRNA reads observed in the DI library correlated with the elevated levels of RBSDV RNA accumulation. Northern blot analysis was carried out to compare RBSDV RNA accumulation levels between singly and doubly-infected *L. striatellus*. For detection we selected RBSDV S5, S7 and S10, which represent RNA segments with significant, slight and no increases of v-siRNAs read numbers in the DI library, respectively (Fig. 3A). RBSDV S5 accumulated to a much higher level in doubly-infected than singly-infected *L. striatellus* while S7 had only a slight increase in doubly-infected planthoppers, and there were no differences in accumulation of S10 between the two samples (Fig. 4A). In northern blot analysis to detect RSV RNA3 and RNA4 accumulation, no difference in RNA accumulation levels was observed between singly and doubly-infected insects (Fig. 4B). Double infection had no significant effect on the viral capsid protein accumulation of either RBSDV or RSV as detected by enzyme-linked immunosorbent assay (ELISA) (Fig. 4C). This result seems consistent with the observation that the accumulations levels of RBSDV S10 and RSV RNA3, which encode the respective outer capsid and nucleocapsid proteins [29]–[31], were not affected by double infection (Fig. 4A, B). Together, these results suggest that the increase of RBSDV siRNA abundance in doubly-infected insects positively correlates with the elevated levels of RBSDV RNA accumulation.

### RBSDV siRNAs were Frequently Generated from Hotspots in the 5′- and 3′-Terminal Regions of Genome Segments

**Figure 5:**
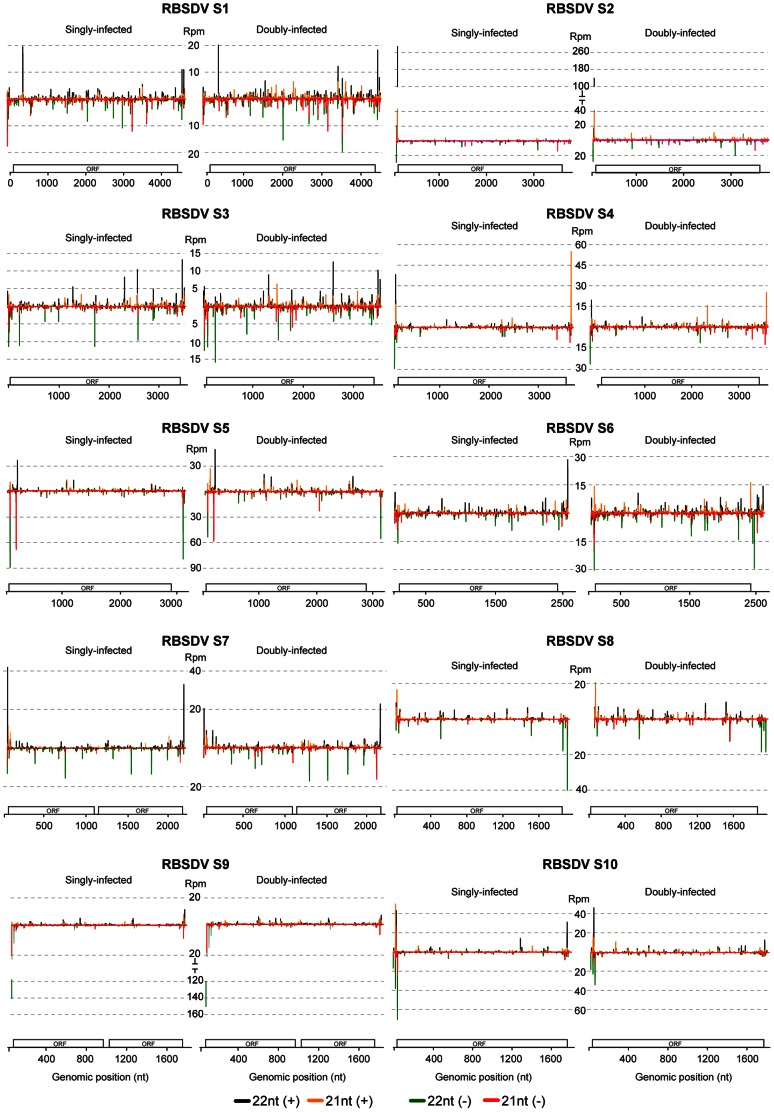
Distribution of RBSDV siRNAs along the ten RNA segments of the RBSDV genome. Schematic representations of open-reading frame (ORF) of each RNA segment are presented. Color coding indicating 21- or 22-nt viral siRNAs derived respectively from the positive (+) and negative (−) genomic strands is presented below the map. Rpm: Reads per million. All reads in this analysis are redundant and normalized.

RBSDV siRNAs of 21- or 22-nt long in the SI_RB library were distributed along the (+)- and (−)-strands of all RBSDV RNA segments, but there were strong siRNA hotspots that mostly mapped to the 5′- and 3′-terminal regions of genome segments (Fig. 5), suggesting that these regions are the preferential targets by the host Dicer. Some siRNA hotspots occurred on both sense and antisense strands (e.g. the 5′-terminal region of S4 and S10) while others were on only one of the strands (asymmetrical) (e.g. the 3′-terminal regions of (+)-strand S4, S6, and S7, and the 5′-terminal regions of (−)-strand S1, S5 and S9) (Fig. 5). Most of the hotspots consist of a cluster of 2 to 7 unique v-siRNAs 21- or 22-nt long. The full length RNA genome segments, or 300-nt at the 5′- and 3′-termini of the (+)- and (−)-strands of each segment were analyzed using the online software UNAFold to identify potential secondary structures that might be responsible for the generation of the siRNA hotspots but no clear relationship between the predicted secondary structures and the siRNA hotspots was found (data not shown). Comparison of RBSDV siRNA distribution profiles between singly and doubly-infected samples showed no substantial difference in distribution pattern. Although v-siRNA reads from the DI library increased in most of RBSDV RNA segments (Fig. 3A), in some RNA segments, the read numbers of siRNA hotspots decreased (e.g. S2, S4, S5, S7, S8 and S10) (Fig. 5). Thus, the RBSDV siRNAs in some viral segments of doubly-infected samples were more evenly distributed than in those of the singly-infected samples.

In RSV, the siRNAs of 21- or 22-nt long from RNA1 (which had the most abundant v-siRNAs) almost saturated the entire segment including the untranslated regions (UTR) (Fig. S1). In RSV RNAs 2, 3 and 4, siRNAs mostly originated from the coding regions and very few from the intergenic regions. The presence of siRNA hotspots was also observed in the RSV genome, although they were less prominent and did not always map to the 5′- and 3′-terminal regions as observed for RBSDV. Furthermore, no obvious difference in distribution profiles of RSV siRNAs between the SI_RSV and DI libraries was observed. Nevertheless, some RSV siRNA hotspots were reduced in read numbers in the DI library (Fig. S1).

### Co-infection with RSV Altered the Distribution of the 5′-terminal Nucleotide of RBSDV siRNAs

**Figure 6:**
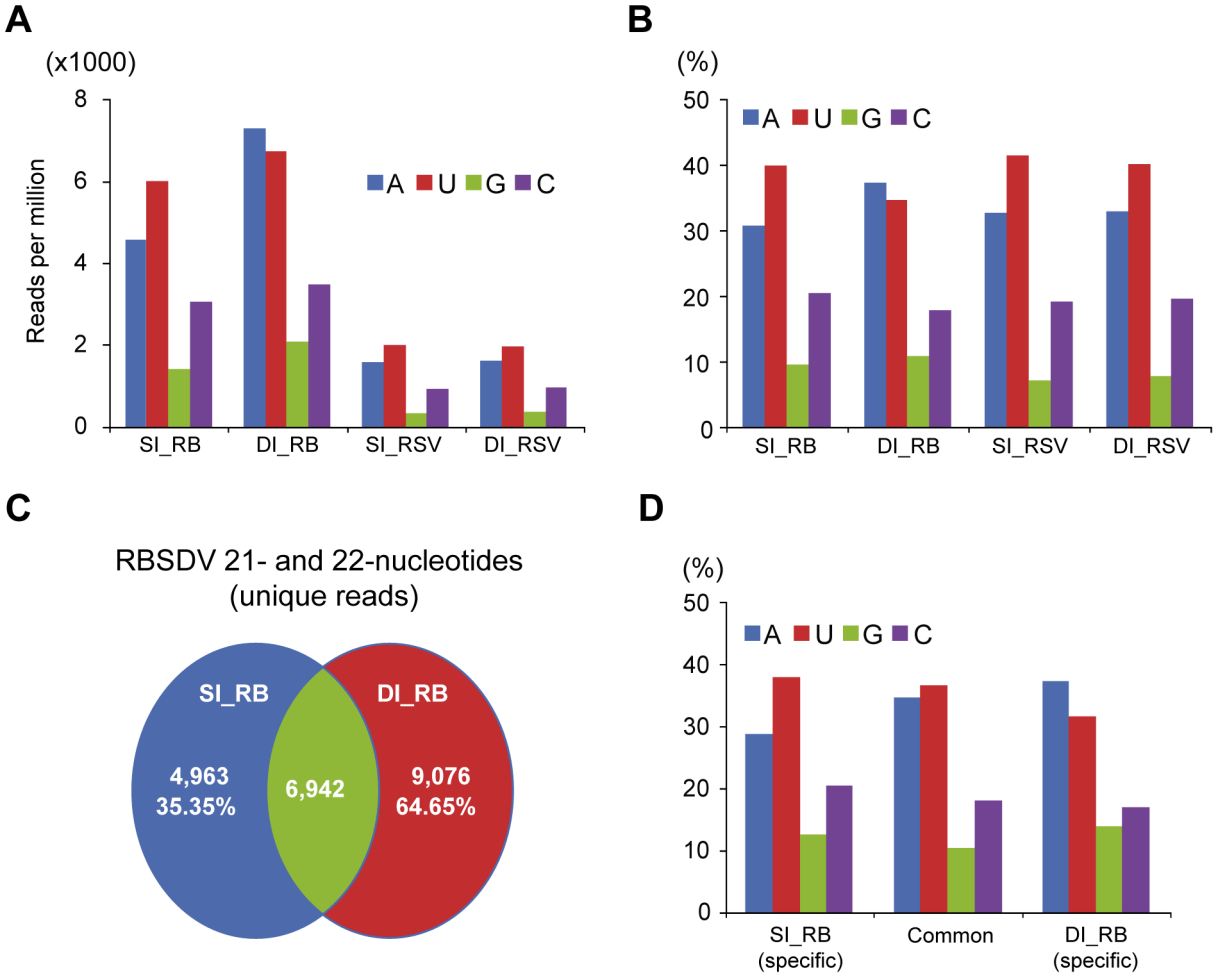
Distribution of the 5′-terminal nucleotide of RBSDV and RSV siRNAs derived from singly (SI_RB or SI_RSV) and doubly-infected (DI) libraries. (**A**) Normalized number. (**B**) Percentage of total viral RNA reads in the corresponding library. (C) Pairwise comparisons of unique RBSDV siRNAs (21- and 22-nt) between singly (SI_RB) and doubly-infected (DI) libraries were done to determine the numbers of viral siRNAs that have specific or shared cleavage sites. (D) Analyses were carried out for 21- and 22-nt unique RBSDV siRNAs that have been grouped according to the analysis presented in C. All reads in this analysis are redundant except (C) and (D) which are unique.

It has previously been reported that the 5′-terminal nucleotide of small RNAs is the key factor that mediates sorting of small RNAs into AGO complexes in plants [32]. Similarly in Drosophila, the 5′-terminal nucleotide of the small RNAs also affects their partitioning between AGO1 and AGO2. Small RNAs binding to AGO2 most frequently begin with C, whereas AGO1-bound small RNAs usually begin with U [33]. The 5′-terminal nucleotide of the RBSDV siRNAs in the SI_RB library and of the RSV siRNAs in both the SI_RSV and DI libraries was most frequently U, followed in order by A, C and G. In contrast, the 5′-terminal nucleotide of the RBSDV siRNAs in the DI library was slightly more likely to be A than U (Fig. 6A and B). The distribution of the 5′-terminal nucleotide of RSV siRNAs was almost identical between the SI_RSV and DI libraries (Fig. 6A and B). Thus co-infection affected the distribution of the 5′-terminal nucleotide of the RBSDV siRNAs but not of the RSV siRNAs. Next, the unique RBSDV siRNAs (21- and 22-nt) in the SI_RB and DI libraries were grouped based on whether they had common or specific cleavage sites as shown in Fig. 6C, and then the distribution of the 5′-terminal nucleotide was analyzed. The result showed that the RBSDV siRNAs generated from specific cleavage sites in doubly-infected insects have a 5′-terminal nucleotide bias toward A (Fig. 6D).

## Discussion

The *Reoviridae* is one of the largest families of viruses, and members have a wide range of hosts across different eukaryotic kingdoms including plants, fungi, insects and mammals [34]. All plant-infecting reoviruses, including RBSDV, can replicate in their insect vectors [4], but their molecular interactions with the host antiviral RNAi pathway remain largely unknown. Because v-siRNAs are the key mediators of the antiviral RNAi response, their identification and characterization provides important clues for the understanding of antiviral responses. Using RBSDV and *L. striatellus* as a virus-insect pathosystem, this study aimed to investigate whether replication of a reovirus in an insect vector induces the generation of viral siRNAs. By analyzing the data derived from deep sequencing of small RNAs, we identified considerable numbers of RBSDV-derived siRNAs generated in *L. striatellus* cells infected with RBSDV (Table 1). Thus, reovirus replication in its insect vector may induce an RNAi-mediated antiviral response.

Compared with the RSV siRNAs that were also analyzed in this study, RBSDV infection generated many more v-siRNAs (Fig. 2B). This difference may be associated with the higher RNA accumulation levels of RBSDV than those of RSV in the cells. The prevalent v-siRNAs were of 21-nt (28–31%) and 22-nt (47–50%) for both RBSDV and RSV. A recent investigation of the RSV-siRNAs in *L. striatellus* is also in agreement with our results [22], whereas previous studies in mosquitoes and Drosophila all found that the 21-nt v-siRNAs were the most abundant [16]–[20]. In Drosophila, DCR1 produces 22-nt microRNAs while DCR2 is responsible for the production of 21-nt siRNAs [35]. It is possible that DCR2 in *L. striatellus* generates two classes of v-siRNAs or perhaps that *L. striatellus* DCR1 and DCR2 are responsible for the production of 22- and 21-nt v-siRNAs, respectively, similar to observations in plants, where multiples Dicers act in concert to produce different sizes of v-siRNAs [36]. Further studies are needed to investigate these possibilities.

Our analyses showed that almost equal amounts of (+)- and (−)- sense RBSDV siRNAs were generated in *L. striatellus* (Fig. 2B), which is similar to the proportions of (+)- and (−)- sense v-siRNAs derived from dsRNA viruses identified in Drosophila [17], suggesting that this is a general feature of v-siRNAs from dsRNA viruses. Our results therefore favor a model that the dsRNA genome or dsRNA RI of RBSDV are the targets of the host Dicer and serve as the major substrates for v-siRNAs production in *L. striatellus*. Moreover, approximately equal proportions of (+)- and (−)- sense v-siRNAs were derived from several ssRNA viruses in Drosophila and mosquitoes, which also suggests that viral dsRNA RI serves as a substrate for v-siRNA production [16]–[20], [37]. In one example, v-siRNAs derived from RNAi suppressor defective *Flock house virus* (FHV, *Nodaviridae*) mutants in Drosophila were almost equally derived from the (+)- and (−)-strands of the RNA genome and a high proportion mapped to the 400-nt at the 5′-terminus, which suggest that this region, formed during the synthesis of (+)-strand RNA, is processed by DCR2 to generate v-siRNAs [16]. The presence of the siRNA hot spots in the 5′- and 3′-terminal regions of RBSDV genome segments may imply that the nascent dsRNAs that form during the synthesis of (+)- or (−)- strand genomic RNA are preferentially targeted by Dicer. However, some segments showed the presence of asymmetrical siRNA hotspots in the 5′- or 3′-terminal regions, suggesting that these regions may highly structured and targeted by Dicer, although no such relation was observed by bioinformatics analysis. Considering the current model of intra-particle viral RNA synthesis of reoviruses, the accumulation of (−) sense RBSDV siRNAs raises the question of how Dicer gains access to the RBSDV (−)-strands RNA or dsRNA in the *L. striatellus* cells. As reovirus infection is also known to induce dsRNA-activated host immune responses such as interferon [38], it seems probable that the dsRNAs are exposed to host antiviral machineries during a certain step of reovirus genome replication. Indeed, in a nondestructive *in situ* analysis using a dsRNA-specific antibody, significant amounts of viral dsRNA were detected in cells infected with a reovirus [39]. Elucidating the precise mechanism by which RNAi machineries sense and make contact with reovirus dsRNA will be an interesting topic for future studies.

Multiple viral infections in the same host commonly occur in plants, fungi and mammals [40]–[42]. Whether these viruses interact synergistically or antagonistically within their host may be crucial to the disease outcome [43]–[45]. In this study, mixed infections of RSV and RBSDV were established in its vector *L. striatellus* under laboratory conditions (Fig. 1). Although the effects of such mixed infections on the performance of *L. striatellus* are still not clear, we observed that mixed infection resulted in increased accumulation of particular RNA segments of RBSDV but not its capsid protein (Fig. 4). Thus, it seems that RSV infection does not enhance the overall RBSDV genome replication but increases the accumulation of specific genome segments. Viral synergism is commonly observed in mixed infections of plant viruses, one of the most well characterized being the mixed infection of potyviruses with unrelated viruses [42]. The stimulatory effects of potyviruses on the accumulation of the second virus is due to the suppression of antiviral RNA silencing by the helper component-proteinase (HC-Pro) silencing suppressor encoded by potyviruses [46], [47]. It is also important to note that the synergistic effect on genome accumulation of a fungal-infecting reovirus (*Mycoreovirus 1*) in mixed infection with *Cryphonectria hypovirus 1* (CHV1; *Hypoviridae*) is mediated by p29, a CHV1-encoded multifunctional protein with an RNA silencing suppression activity [44]. Previously, the NS3 and p2 (NS2) proteins of RSV were shown to have RNA silencing suppression activity in plants [48], [49]. RSV NS3 has an ability to bind siRNAs [49], which is similar to the activity of NS3 encoded by *Rice hoja blanca virus* (*Tenuivirus*) [50]. Given the ability of RSV to suppress the RNAi pathway through virally-encoded suppressor proteins it is tempting to suggest that strong inhibition of the antiviral RNAi response by RSV contributes to the elevated levels of RBSDV RNA accumulation in *L. striatellus*. In double infections, there were more v-siRNAs derived from RBSDV S5 and S7 that had elevated levels of RNA accumulation, but no changes in the abundance of v-siRNAs derived from RBSDV S10, which showed no increase in RNA accumulation (Fig. 3 and 4). This result suggests that enhanced replication of RBSDV RNA segments provides more dsRNA substrate for biosynthesis of v-siRNAs. In this experiment, the insects were first infected with RSV. Further experiments are needed to determine whether a similar phenomenon occurs if the insects are first infected with RBSDV. Notably, a more marked increase of RBSDV siRNAs in doubly-infected insects was observed for v-siRNAs that had a 5′-terminal A, U or G than for those with C (Fig. 6A). The siRNA duplex with a 5′-terminal C is the most efficiently loaded into AGO2, an AGO component required for the antiviral defence [33]. It is therefore possible that there is no significant increase of AGO2-loaded v-siRNAs in doubly-infected insects, and therefore the increase of RBSDV siRNA abundance might not strongly affect RBSDV RNA accumulation.

## Materials and Methods

### Insect Culture, Virus and Host Plant

The populations of virus-free and RSV-infected *L. striatellus* were provided by Tong Zhou (Institute of Plant Protection, Jiangsu Academy of Agricultural Sciences, China). Insects were reared on susceptible rice (cv. Wuyujing No. 3) in climate-controlled rooms at 26±1°C, with a photoperiod of 16 h light: 8 h darkness and 70±10% relative humidity. The RSV-infected *L. striatellus* population was sustained for more than 20 generations and the infection ratio (around 50%) was monitored every 3–5 generations by RT-PCR. The RBSDV-infected rice plants with typical symptoms used in this study were collected from fields in Shandong Province, China (provided by Qisong Zhu of Shandong Academy of Agricultural Sciences, China) and the identity of the virus was confirmed by RT-PCR.

### Virus Acquisition and *L. striatellus* Collection

Approximately 1000 of the 2^nd^ instar nymphs derived from the RSV-infected *L. striatellus* population were released onto RBSDV-infected rice plants. After a 2 days acquisition access period, surviving nymphs were transferred to healthy rice seedlings for the insects to pass the latent period. After around 20 days feeding on the healthy rice, newly-emerged *L. striatellus* adults were collected and divided into four groups according to their gender and wing form (macropterous males, brachypterous males, macropterous females and brachypterous females) by visual observation. In total, about 700 insects were collected with glass tubes and stored at −80°C immediately after observation of the gender and wing form. The 419 insects were used for RNA extraction.

### Total RNA Extraction and RT-PCR

Total RNAs were extracted from individual insects with Trizol (Invitrogen, USA) according to the manufacturer’s instructions. Approximately 0.2 µg of total RNA was obtained from each insect and then 0.02 µg of the RNA was used for virus detection using the One Step RT-PCR Kit (TOYOBO, Japan) according to the manufacturer’s instructions. For detection of RSV, the primers (which are specific to RNA3) were: RSV-F (5′-gcctcatcctcgaagaactcct-3′) and RSV-R (5′-gccagccactctagctgatttg-3′). For detection of RBSDV, the primers (which are specific for S1) were: RB-F (5′-acccagtcaagacgctcagt-3′) and RB-R (5′-ctgttcccgccatagacact-3′). Based on the RT-PCR results, the remaining RNAs from each insect were pooled into four groups: those with RSV, RBSDV, RSV plus RBSDV or neither virus. Each pool of RNAs was derived from 60 insects with the same ratios of gender and wing form, and then used for deep sequencing. RNA integrity and quality were assessed by denaturing agarose gel electrophoresis and 2100 Bioanalyzer (Agilent, USA). The remaining RNAs were also used for Northern blot.

### Northern Blot and ELISA Analyses

Ten µg of total RNA of each sample was used for Northern blot analysis. The blots were hybridized with digoxigenin-labeled DNA probes specific for RSV RNA3 (nt 1794 to 2242) and RNA4 (nt 56 to 508) as well as RBSDV S5 (nt 2378 to 3073), S7 (nt 1420 to 1856) and S10 (nt 103 to 610). DNA probes were prepared using the PCR DIG Probe Synthesis Kit (Roche, Germany). RNA gel electrophoresis and blotting were carried out as described previously [51]. The hybridization conditions and detection of mRNAs were done as described in the DIG Application Manual supplied by Roche.

Double-antibody sandwich ELISA was carried out following the method described previously [52] using monoclonal antibodies specific for RBSDV outer capsid (P10) and RSV nucleocapsid (NSvc3) proteins kindly provided by Jianxiang Wu (Zhejiang University, China). Absorbance values at 405 nm were recorded with a Spectramax M5 plate reader (Molecular Devices, USA). Six individual insects of each group (SI_RSV, SI_RB, DI and VF) were analyzed. A Student’s t-test was used for paired analysis and a value of *P*<0.05 was considered to be significant. Statistical analysis was performed using the SPSS software package for Windows (Chicago, USA).

### Small RNA Deep Sequencing and Bioinformatics Analysis

The cDNAs of small RNA libraries were prepared by using the Illumina TrueSeq Small RNA Sample Preparation Kit (Illumina, San Diego, CA, USA). Briefly, the total RNA (5 µg) was resolved on a denatured 8% polyacrylamide gel electrophoresis (PAGE), and then the fraction of 18 to 30 nt small RNAs was purified. The isolated small RNA were sequentially ligated to a 3′- adapter using T4 RNA ligase 2 (New England Biolabs, Ipswich, MA, USA) and a 5′- adapter using T4 RNA ligase (New England Biolabs, Ipswich, MA, USA). The ligation products were reverse transcribed using SuperScript II Reverse Transcriptase (Life Technologies, Gaithersburg, MD, USA) and amplified with 12 PCR cycles. A 6% polyacrylamide gel was used to purify the amplification products, which then were sequenced using the Illumina HiSeq2000. Raw datasets of the small RNA were analyzed following the pipeline described previously [18], [21], [22]. MirDeep2 [53] and home-made perl scripts were used during the analysis. In brief, adaptor sequences were trimmed and small RNA reads without an identifiable linker were removed. The remaining reads were filtered by length and the reads of >30-nt or <18-nt were discarded. To identify v-siRNA, processed reads from the four libraries were mapped to the RSV (NCBI accession no: PRJNA14795) and RBSDV genomes (NCBI accession no: PRJNA14790) using Bowtie software (http://bowtie-bio.sourceforge.net). To facilitate comparisons across the four libraries, viral v-siRNA read numbers were scaled to ‘reads per million’ (rpm) based on the total small RNA read numbers of the corresponding library. Raw sequences of RBSDV and RSV small RNAs derived from singly and doubly-infected *L. striatellus* libraries have been deposited in NCBI Sequence Read Archive (SRA; http://www.ncbi.nlm.nih.gov/sra) under the accession number of SRP019775. RNA secondary structures were predicted by the UNAFold web server (http://mfold.rna.albany.edu/) with default parameters. Downstream analyses were carried out using home-made perl scripts.

## Supporting Information

Figure S1 **Distribution of RSV siRNAs along the four RNA segments of the RSV genome.** Schematic representations of open-reading frame (ORF) of each RNA segment are presented. Color coding indicating 21- or 22-nt viral siRNAs derived respectively from the positive (+) and negative (−) genomic strands is presented below the map. Rpm: Reads per million. All reads in this analysis are normalized and redundant.
(TIF)
