# Supplementary figures and images for: Characterization of Rice Black-Streaked Dwarf Virus- and Rice Stripe Virus-Derived siRNAs in Singly and Doubly Infected Insect Vector Laodelphax striatellus
Source: PLoS One. 2013 Jun 11;8(6):e66007. doi: 10.1371/journal.pone.0066007 (PMC3679040; doi:10.1371/journal.pone.0066007)

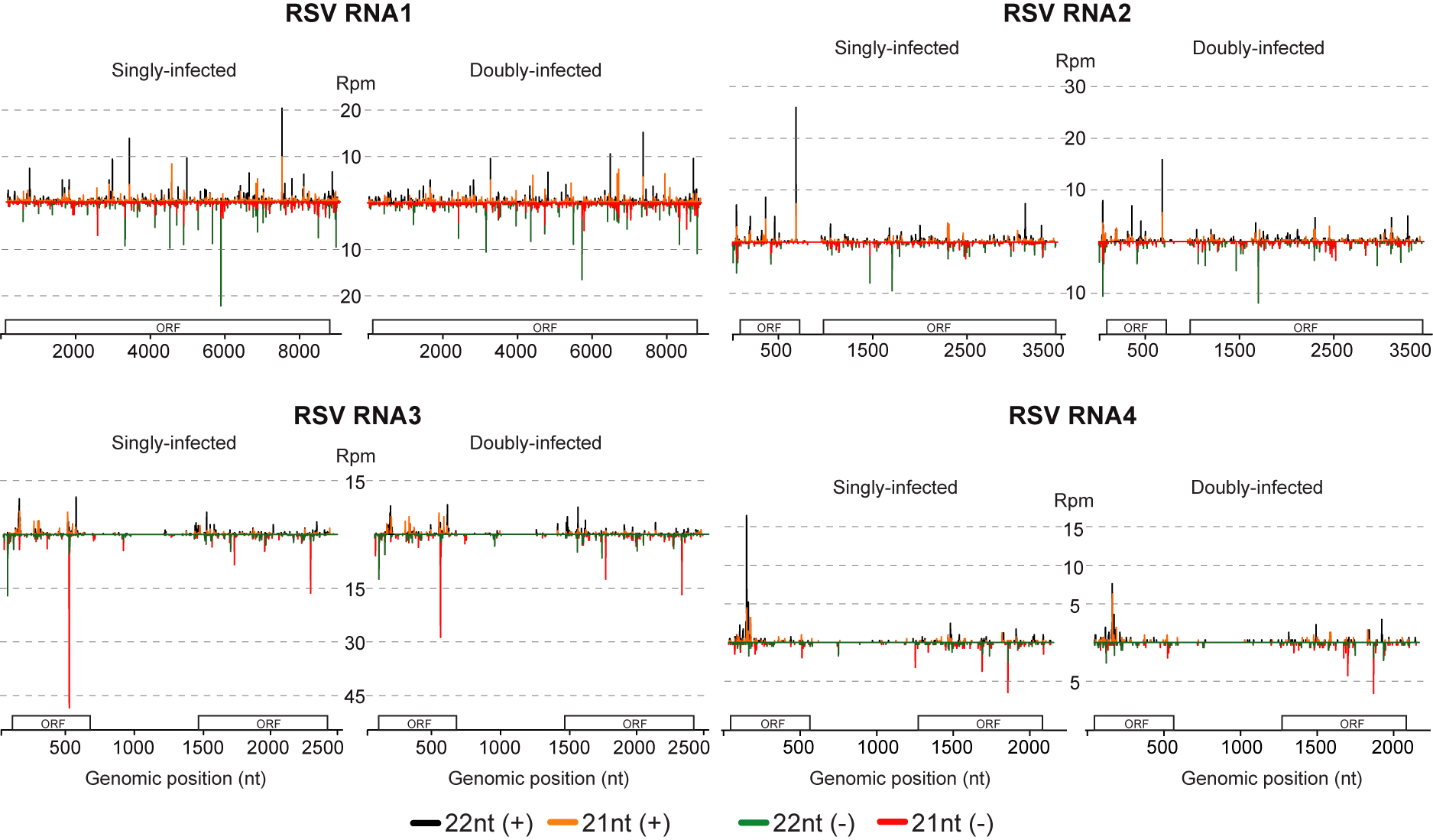

Supplement: Figure S1 — Distribution of RSV siRNAs along the four RNA segments of the RSV genome. Schematic representations of open-reading frame (ORF) of each RNA segment are presented. Color coding indicating 21- or 22-nt viral siRNAs derived respectively from the positive (+) and negative (−) genomic strands is presented below the map. Rpm: Reads per million. All reads in this analysis are normalized and redundant. (TIF) [file pone.0066007.s001.tif]
